# Nitrogen as a Probe Molecule for the IR Studies of the Heterogeneity of OH Groups in Zeolites

**DOI:** 10.3390/molecules26206261

**Published:** 2021-10-16

**Authors:** Łukasz Kuterasiński, Mariusz Gackowski, Jerzy Podobiński, Dorota Rutkowska-Zbik, Jerzy Datka

**Affiliations:** Jerzy Haber Institute of Catalysis and Surface chemistry, Polish Academy of Sciences, ul. Niezapominajek 8, 30-239 Kraków, Poland; mariusz.gackowski@ikifp.edu.pl (M.G.); jerzy.podobinski@ikifp.edu.pl (J.P.); dorota.rutkowska-zbik@ikifp.edu.pl (D.R.-Z.)

**Keywords:** zeolites, acidity, heterogeneity of OH groups, probe molecules, nitrogen

## Abstract

One of the methods of IR studies of the heterogeneity of Si–OH–Al groups in zeolites is the investigation of the frequency shift of the band of free OH bands restored upon the adsorption of ammonia and subsequent desorption at increasing temperatures. We extended this method by following the shift of the band of the OH group interacting by hydrogen bonding with nitrogen. The advantage of nitrogen, compared with CO, which has been commonly used as a probe molecule in studies on hydrogen bonding, is that for nitrogen the frequency shift is smaller than for CO and therefore there is no overlapping of shifted OH band with the bands of ammonium ions. For zeolites NaHY, HMFI, and HBEA, the frequency shift of IR bands of both free and hydrogen-bonded Si–OH–Al with the increase of ammonia desorption temperature evidences the heterogeneity of these hydroxyls. On the other hand, in zeolite HFAU of Si/Al = 31, Si–OH–Al were found to be homogeneous. Heterogeneity of OH groups may be explained both by the presence of Si–OH–Al of various number of Al near the bridge and of Si–OH–Al of various geometry.

## 1. Introduction

Zeolites are microporous solids characterized by many unique properties, which are responsible for high acidity, shape selectivity, and thermal stability. Due to the presence of acid active sites, this group of aluminosilicates can be used in many catalytic industrial processes, such as cracking, esterification, alkylation, isomerization, the production of fine chemicals, and for environmental protection [1,2,3,4,5,6,7,8,9].

Due to the wide application of zeolites as catalysts in oil refineries and in many processes in “fine chemistry”, knowledge of the acidity of zeolites is crucial for catalysis; this must include the determination of the concentration of both Bronsted and Lewis sites, the characterization of their acid strength, and the diagnosis of the problem of heterogeneity. Studies on the problem of heterogeneity are an important part of our knowledge on the acidity of zeolitic catalysts. Therefore, detailed studies of the acidic properties of zeolites have been the subject of intensive study. Since 1973, Jacques Védrine played an important role in this field by publishing many important research papers and reviews, including information on the most useful techniques for zeolite acidity characterization [10,11]. Among known techniques, IR spectroscopy seems to be the most efficient method for acidity studies.

Using IR spectroscopy, protonic acidy can be determined both in the presence and absence of probe molecules, while Lewis acid sites can be investigated only with the application of probe molecules. Interestingly, for the research on the Brønsted acid sites with the use of probe molecules, Raman spectroscopy can also be applied [12]. The type of applied probe molecule should be adapted to the investigated system. Lercher et al. [13], Paukshtis and Yurchenko [14], and Knözinger [15] have defined the criteria for a proper choice of probe molecule for the measurement of the acidity of solid materials. First of all, the probe molecule should be characterized by dominating basic properties and should be able to distinguish Brønsted and Lewis acid sites with simultaneous differentiation of the strength of acid sites. Furthermore, the used probe molecules should have sizes comparable to the reactants used in catalytic performance due to the minimization of the effects of structure constraints.

Based on literature data, many probe molecules were reported as probe molecules for the characterization of zeolite acidity. The most common probe molecules were pyridine, ammonia, and CO. The results obtained with these three probe molecules were presented in hundreds of papers. The adsorption of ammonia on aluminosilicate catalyst was realized for the first time by Mapes and Eischens in 1954 (almost 70 years ago!) [16], and it was the first application of IR spectrometry to the problems of catalysis. Ammonia is a hard Lewis base that is small. Chemisorbed NH_3_ creates two different forms. The protonated form (NH_4_^+^) gives rise to bands near 1450 and 3130 cm^−1^, whereas NH_3_ coordinated to Lewis acid sites demonstrates bands near 1630 and 3330 cm^−1^. The disadvantage of ammonia in quantitative IR studies of sites concentration is the formation of dimers N_2_H_7_^+^ at higher loadings [17].

Pyridine can be classified as a weaker base than ammonia, if we consider pKa value determined in the liquid phase. However, gas-phase basicity indicates that pyridine should be the stronger base than ammonia. Pyridine was used for the first time by Parry et al. at 1963 [18]. Pyridine undergoes protonation more easily than ammonia, and pyridinium ion (PyH^+^) is thermally more stable than NH_4_^+^. Pyridine is also a relatively hard base. The band at ca. 1545 cm^−1^ is attributed to pyridinium ions (PyH^+^), while the band at ca. 1450 cm^−1^ is due to pyridine adsorbed on Lewis acid sites. Both ammonia and pyridine are commonly used in the quantitative analysis of Brønsted and Lewis acidity. It should be mentioned that the first quantitative study of zeolites acidity with pyridine, ammonia, and piperidine was realized by Hughes and White in 1967 [19]. Pyridine was also used for quantitative determination of concentration of sites in “pure” zeolites and zeolites containing various deposits such as coke [20,21,22]. It was found that strongly basic and flat pyridine molecule can react with protonic sites “covered“ by coke.

Pyridine and ammonia differ significantly in terms of molecule size. Ammonia reacts with all the acid sites, even in small cages and narrow pores. Pyridine detects only the sites in channels larger than ca. 0.53 nm, i.e., the sites accessible to most of reactants. If zeolite has both large and narrow channels, the application of the two probe molecules ammonia and pyridine makes the determination of concentrations of sites in each kind of channel possible. This was done for mordenites in [23], in which the concentration of sites in 8- and 12-ring channels was determined separately.

In turn, CO can be used for the characterization of the strength of acidic hydroxyls via the comparison of the values of frequency shifts of the bands of hydroxyls interacting by hydrogen bonding with CO. Moreover, the frequency of the C≡O band at ca. 2160–2180 cm^−1^ increases with the acid strength of hydroxyls, so the position of this band also affects the strength of acidic hydroxyls.

Carbon monoxide is also a very good probe molecule for the study of the status of Lewis acid sites. CO is able to distinguish different types of Lewis acid sites: strongly acidic sites produced by dehydroxylation of acidic hydroxyls (CO band 2220–2230 cm^−1^) and less acidic sites that are extra-framework Al species (CO bands around 2190 cm^−1^). Such an analysis of CO frequency was helpful in the discussion of changes of status of Al in hierarchical desilicated zeolites Y [24]. The adsorption of CO was also used for studies of properties of cations (e.g., Na^+^, K^+^, Ca^2+^, Cu^+^, Co^2+^, Ni^2+^, and Fe^2+^).

Another example of probes were organic molecules, such as alkanes, alkenes, benzene, acetonitrile, ethers, acetone, and aldehyde. Alkanes (e.g., n-hexane) interact with hydroxyls by a “permanent dipole-induced dipole” interaction, and red frequency shift may be taken as the measure of their acid strength [13,25,26]. The information on acid strength may also be obtained by comparing the values of frequency shifts of hydroxyls mixing hydrogen bonding with alkenes [27,28,29,30,31], benzene [32,33,34], or other aromatic hydrocarbons. Due to its small molecular size, ethane can reach sites not accessible to bulky benzene. Acetonitrile and perdeuterated acetonitrile were also used for studies on the acidity of solids [35,36,37]. The C≡N shifts by 10–30 cm^−1^ when interacting with OH groups and by 30–60 cm^−1^ when interacting with Lewis acid sites. Lavalley et al. [38] used deuterated dimethyl ether (CD_3_-O-CD_2_H) to measure the acidity of solids. The C-H stretching was sensitive regarding the acidity of the adsorption site. Ketones [36,39,40,41] and aldehydes [42,43] were also used as probe molecules. The C=O frequency (similarly as C≡N frequency in nitriles) depends on the acidity of adsorption sites.

Important information on the accessibility of acid sites for bulky molecules was obtained by IR studies of bulky probe molecules such as pivalonirile, 2,6 ditertbutylpyridine, 2,4 dimethylquinolone, 2,4,6-trimethylpyridine, or di-tert-butylether [44,45,46,47,48,49,50,51]. These bulky molecules, which cannot penetrate zeolites channels, were also used to distinguish acid sites on external surfaces of zeolite crystals or on mesopore surfaces and inside channels. Some of these bulky molecules (pivalonirile; 2,6 ditertbutylpyridine; and 2,4 dimethylquinolone) were also applied in quantitative IR studies of the concentration of sites on external surfaces of zeolites and MCM-36 [52], and on mesopore walls of hierarchical zeolites [53].

The application of small molecules, such as N_2_, O_2_, CH_4_, Ar, and H_2_, as probe molecules for acidity measurements has also been reported. The adsorption of nitrogen on cationic forms of zeolites was studied. The authors [54,55,56] reported the appearance of N≡N stretching band, even though this vibration was IR-inactive in free molecules. The interaction of nitrogen with the adsorption site changed the molecular symmetry. The DFT calculations [57] suggested the end-on configuration of nitrogen hydrogen bonded with zeolitic acidic OH groups. The OH frequency shift was ca. 100 cm^−1^ for zeolite HMFI [58]. A similar frequency shift was observed for zeolite HY [56].

Oxygen (similarly to nitrogen) forms hydrogen bonding with zeolitic hydroxyls, as reported by Makarova [30] and by Gribov et al. [56], but in this case, the frequency shift was smaller (ca. 50 cm^−1^) than for nitrogen (ca. 100 cm^−1^). The O=O vibration also became IR-active, and the band at ca. 1550 cm^−1^ appeared. Argon interacts with OH groups by a “permanent dipole-induced dipole” interaction (similarly as alkenes), but the frequency shift is much smaller (ca. 30 cm^−1^) [56]. The adsorption of hydrogen in zeolites was followed by several authors, e.g., Kazansky [59] and Gribov [56]. The H-H stretching became IR-active, and the bands around 4000 cm^−1^ appeared. Hydrogen adsorption requires very low temperatures (ca. 80 K), so not many laboratories are able to realize such studies.

**Figure 1:**
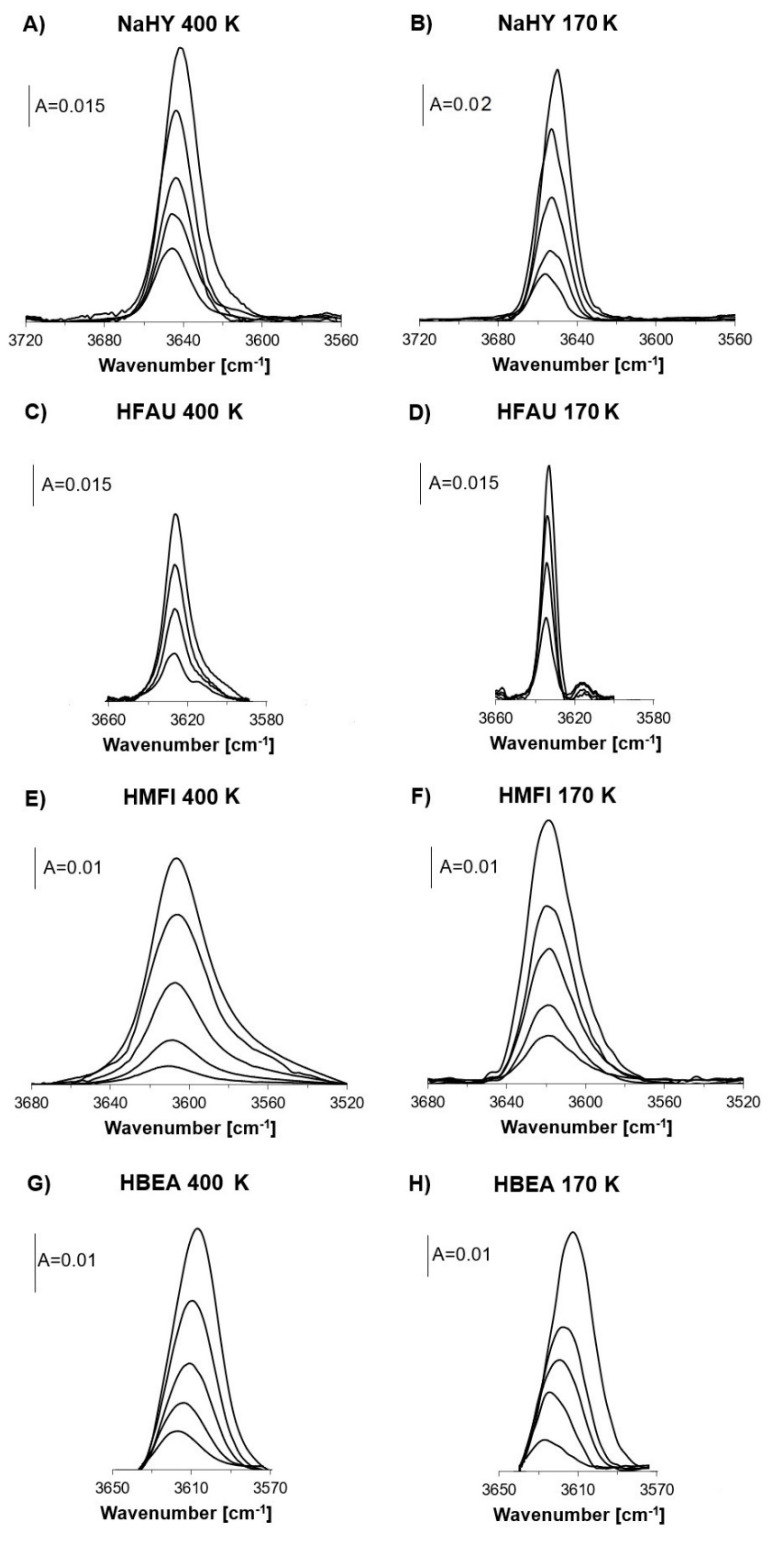
IR spectra of free Si–OH–Al groups in zeolites NaHY (**A**,**B**), HFAU (**C**,**D**), HMFI (**E**,**F**), and HBAE (**G**,**H**), recorded at 170 K and 400 K. Top spectra are spectra of zeolites before ammonia sorption. All other spectra were recorded upon the sorption of ammonia and subsequent desorption at increasing temperatures. Desorption temperatures from bottom to top: NaHY 418, 433, 443, and 458 K; HFAU 473, 483, and 505 K; HMFI 473, 483, 503, and 518 K; and HBEA 443, 463, 483, and 503 K.

This study concerns the problem of the heterogeneity of Si–OH–Al groups in zeolites (i.e., the problem of the coexistence of hydroxyls of various acid strengths). Several methods of solving the problem of heterogeneity were applied. One of them was the neutralization of all the hydroxyls and desorption step-by-step at increasing temperatures. If Si–OH–Al are heterogeneous, less acidic ones of higher stretching frequency release ammonia at lower temperatures, compared to more acidic hydroxyls of lower frequency. As the result, the band of free acidic hydroxyls restores shifts to lower frequencies with the increase of desorption temperature. On the other hand, no such frequency shift is observed for homogeneous Si–OH–Al. In this study, we modified this method by the adsorption of nitrogen on zeolite upon the desorption of ammonia. Nitrogen interacts by hydrogen bonding with hydroxyls, which is restored by ammonia desorption. The frequency shift Δν_OH…N2_ increases with the acid strength, so it is expected that the band of Si–OH–Al groups interacting by hydrogen bonding will also shift to lower frequencies, i.e., Δν_OH…N2_ will increase with ammonia desorption temperature. Nitrogen is a suitable probe molecule for this kind of experiment, and it is better than, CO which has been commonly used in studies on the hydrogen bonding of OH groups. The band of Si–OH–Al interacting with CO appears at ca. 3300 cm^−1^, i.e., in the region in which NH_4_^+^ bands are present (this is seen in Figure 1); moreover, CO forms hydrogen bonding with N-H in NH_4_^+^, which makes the spectrum illegible. The band of zeolitic OH interacting with N_2_ appears at ca. 3500 cm^−1^, i.e., in the region in which NH_4_^+^ bands are absent. The frequency shift of OH…N_2_ band (similarly as OH…CO) increases with the acid strength of OH [60,61].

Another method of the studies of heterogeneity is the analysis of the broad band of H hydrogen bonded with aromatic hydrocarbons [62,63]; however, because of the Fermi resonance, such an analysis is difficult [58].

Four zeolites were studied: NaHY (Si/Al = 2.5), HFAU (Si/Al = 31), HMFI (Si/Al = 20), and HBEA (Si/Al = 25). These zeolites differ in the number of Al near OH and in the geometry of Si–OH–Al bridge. Si–OH–Al in zeolite NaHY have various Al in the vicinity of Si–OH–Al but the same bridge angle in all Si–O_1_H–Al. On the other hand, in HFAU all the Si–O_1_H–Al have the same number of Al near the bridge and the same bridge geometry. Both HMFI and HBEA contain Si–OH–Al of various numbers of Al near the bridge, and various geometries. As the acid strength of Si–OH–Al may depend both on the number of Al near the bridge and on bridge geometry, in some of these zeolites Si–OH–Al may be homogeneous and in others heterogeneous. As the spectra of zeolites NaHY show two intensive bands of Si–O_1_H–Al and Si–O_3_H–Al at ca. 3640 and 3550 cm^−1^, respectively, and because the 3550 cm^−1^ band overlaps the OH…N_2_ one, we used zeolite NaHY of very low exchange degree (11%) in which the 3550 cm^−1^ OH band is absent. As mentioned, we studied the heterogeneity of Si–OH–Al groups by the adsorption of nitrogen on zeolites in which ammonia was adsorbed and next desorbed step-by-step at various temperatures.

## 2. Results

### 2.1. Free OH Groups

The spectra of free Si–OH–Al groups in zeolites: NaHY (Si/Al = 2.5), HFAU (Si/Al = 31): HMFI (Si/Al = 20), and HBEA (Si/Al = 25), and in which ammonia was preadsorbed and subsequently desorbed at increasing temperatures (in the range 418–503 K) are presented in Figure 1. The spectra of OH groups in the same zeolites without ammonia are given too. The spectra recorded at both 400 K and 170 K are presented. The frequencies of Si–OH–Al are given in Table 1. For NaHY and HBEA, the Si–OH–Al band shifts to lower frequencies by 5–10 cm^−1^, suggesting heterogeneity of these OH groups. In the case of dealuminated HFAU, no frequency shift is observed. For HMFI, the small frequency shift (4 cm^−1^) in the spectra recorded at 400 K (Figure 1E) and no frequency shift are seen at 170 K (Figure 1F, and Table 1). More information on heterogeneity and homogeneity of acidic hydroxyls in these zeolites will be obtained by following the spectra of these hydroxyls interacting with nitrogen by hydrogen bonding.

### 2.2. OH Groups Interacting with Nitrogen

The most valuable information on the acid strength of hydroxyls in zeolites (and on other solids) may be obtained by following hydrogen bonding of these hydroxyls with electrondonor probe molecules. It is well known that the frequency shift accompanying hydrogen bonding increases with the acid strength of OH. In our study, the IR band of OH groups engaged in hydrogen bonding with nitrogen was studied in zeolites, in which ammonia was preadsorbed and subsequently desorbed at increasing temperatures. It was expected that (similarly to free OH groups) for heterogeneous OH groups, the IR band of hydrogen-bonded hydroxyls would shift to lower frequencies with the ammonia desorption temperature. As the Δν of OH hydrogen bonded with electrondonor molecules is more sensitive to the variation of acid strength of hydroxyls than the Δν of free OH groups, the frequency shift of IR band of hydrogen bonded OH should be more significant than the frequency shift of free OH groups. This will provide the information on heterogeneity of Si–OH–Al groups, which is not only supplementary to those obtained by following the free OH band but also more unequivocal.

**Figure 2:**
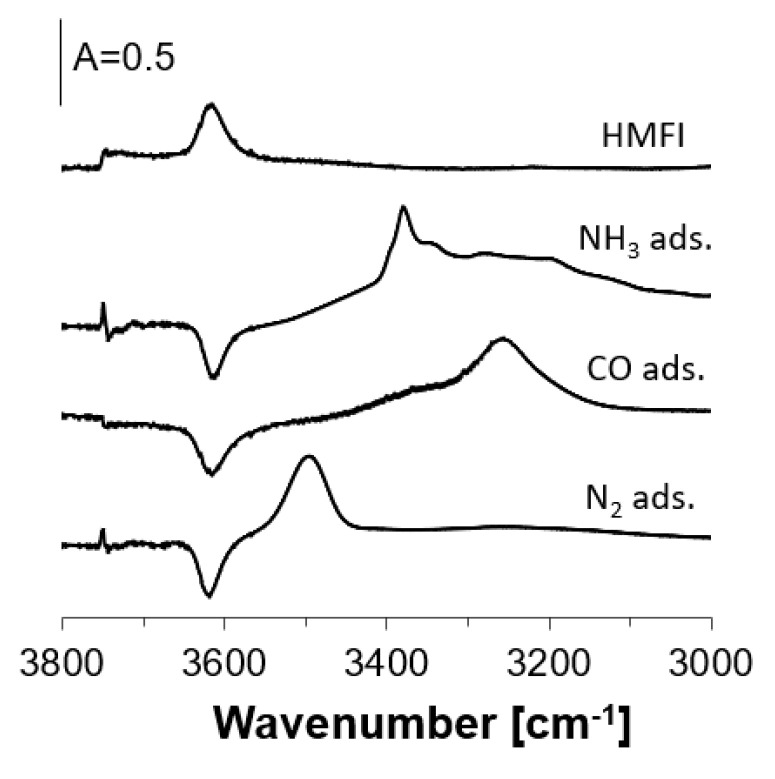
IR spectra of OH groups in zeolite HMFI and MFI with adsorbed ammonia, CO, and N_2_. All the spectra were recorded at 170 K.

As mentioned in Introduction, we used nitrogen as a probe molecule for the study of hydrogen bonding of OH in zeolites with preadsorbed ammonia. Even though CO is now the most useful and most commonly used probe molecule for the studies of acid strength of zeolitic hydroxyls, it is useless for zeolites containing NH_4_^+^ because the band of Si–OH–Al groups interacting with CO appears in the same region (ca. 3300 cm^−1^) in which the bands of NH_4_^+^ are present. This is seen in Figure 2, in which the spectrum of ammonium ions in zeolite HMFI, the spectrum of Si–OH–Al interacting with CO, and the spectrum of Si–OH–Al interacting with N_2_ are presented. Both CO and N_2_ were sorbed at ca. 170 K. The spectrum of free OH groups is shown too. Nitrogen is better probe molecule for Si–OH–Al in zeolite with preadsorbed ammonia, because the frequency shift of Si–OH–Al interacting with hydrogen bond with nitrogen is ca. 100 cm^−1^ and the shifted band does not overlap the bands of NH_4_^+^ ions.

**Figure 3:**
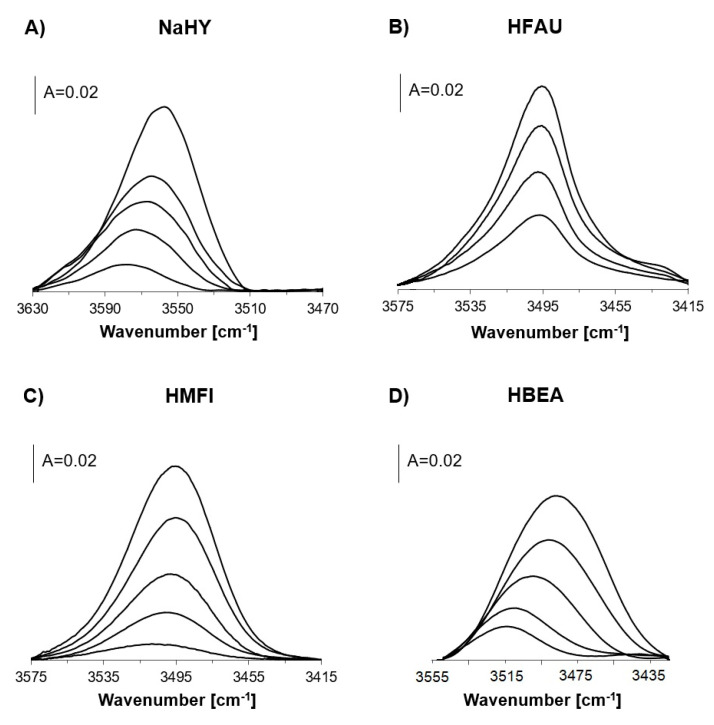
The IR spectra of Si–OH–Al groups in zeolites NaHY (**A**), HFAU (**B**), HMFI (**C**), and HBAE (**D**) interacting with nitrogen at 170 K. Top spectra were recorded upon nitrogen sorption in zeolites without ammonia. All other spectra were recorded upon nitrogen sorption in which ammonia was sorbed and subsequently desorpbed at increasing temperatures. Desorption temperatures from bottom to top: NaHY 418, 433, 443, 458 K; HFAU 473, 483, 505 K; HMFI 473, 483, 503, 518 K; and HBEA 443, 463, 483, and 503 K.

The spectra of Si–OH–Al groups in zeolites NaHY, HFAU, HMFI, and HBEA interacting with N_2_ in zeolites in which ammonia was preadsorbed and subsequently desorbed at increasing temperatures are presented in Figure 3, and the values of Δν_OH…N2_ are given in Table 1. In the case of HMFI, HBEA, and HY, the band of OH…N_2_ shows blue shift (Δν_OH…N2_ increases), indicating heterogeneity of Si–OH–Al groups, whereas for HFAU no such shift is observed, evidencing homogeneity of acidic hydroxyls. For OH…N_2_ bands, the frequency shifts are more significant than for free OH, so the conclusions are more unequivocal.

The spectra of nitrogen sorbed in our zeolites show weak and narrow band of N≡N band around 2330 cm^−1^ (spectra not shown). The N≡N stretching, which is IR-inactive in free molecules, became active, and the DFT calculations [57] suggested the end-on configuration of nitrogen connected with zeolitic acidic OH via hydrogen bonds.

## 3. Discussion

The reason why Si–OH–Al groups in some zeolites are heterogenous and in some others homogeneous may be explained by considering which factors affect the acid strength of these hydroxyls. The acid strength depends mostly on two factors: the number of AlO_4_^−^ in the vicinity of Si–OH–Al, and the bridge geometry. The environment of OH group may be represented by the following formula: (AlO)_n_(SiO)_3-n_Si–OH–Al(OSi)_3_. The higher the n number is, the lower the acid strength of Si–OH–Al will be. This was evidenced by quantum chemical calculations [64,65,66] and by many experimental facts (the acidity increases in the order zeolite X < zeolite Y < dealuminated Y). Another factor influencing the acid strength is the Si–OH–Al bridge angle as well as Si-O and Al-O distances. This was proved by quantum chemical calculations of Beran [67,68] and Sauer et al. [69,70].

**Figure 4:**
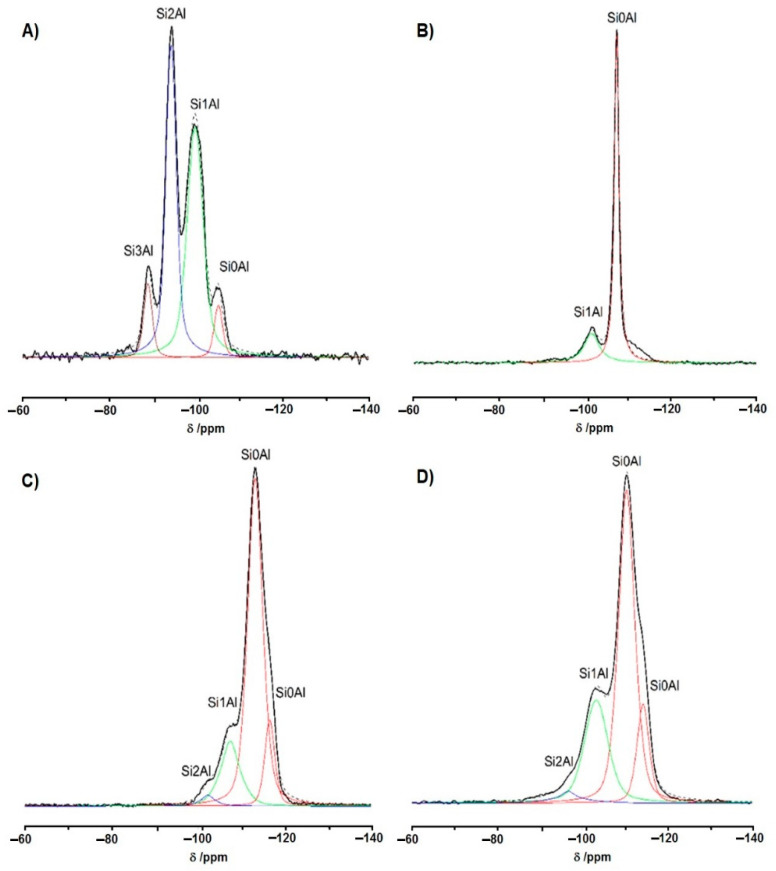
The 29Si MAS NMR spectra of zeolites NaY (**A**), HFAU (**B**), HMFI (**C**), and HBEA (**D**). The spectra are normalized to the same intensity of the highest signal.

The information on the number of Al atoms (and therefore on the number of AlO_4_^−^) near the bridge may be obtained from ^29^Si MAS NMR spectra (Figure 4). The spectrum of zeolite Y (Si/Al = 2.5) (Figure 4A) shows four ^29^Si signals of Si(0Al), Si(1Al), Si(2Al), and Si(3Al). As Si(0Al) cannot form bridging hydroxyls, three kinds of Si–OH–Al of various acid strengths may be present. They may be represented by the following formula: (SiO)_3_Si–OH–Al(OSi)_3_, (AlO)(SiO)_2_Si–OH–Al(OSi)_3,_ and (AlO)_2_(SiO)Si–OH–Al(OSi)_3_. The most acidic are (SiO)_3_Si–OH–Al(OSi)_3_, and the less acidic are (AlO)_2_(SiO)Si–OH–Al(OSi)_3_. This explains the heterogeneity of Si–OH–Al groups in zeolite Y.

Different situation is observed in strongly dealuminated zeolite HFAU of Si/Al = 31. The ^29^Si MAS NMR spectrum of this zeolite (Figure 4B) shows two signals: intensive one of Si(0Al) and less intensive one of Si(1Al). The signal of Si(2Al) is absent. Therefore, only one kind of strongly acidic hydroxyl represented by the formula (SiO)_3_Si–OH–Al(OSi)_3_ exists. As mentioned above, the acid strength of Si–OH–Al also depends on the geometry of bridge. The acidic hydroxyls vibrating at ca. 3650 cm^−1^ (Si–O_1_H–Al) have the same geometry; therefore, Si–OH–Al in HFAU (of high Si/Al) are homogeneous. According to the data presented in Figure 1C,D and Figure 3B as well as date given in Table 1, the Si–OH–Al in HFAU are indeed homogeneous, as expected from the analysis of NMR spectrum.

According to the data presented in Figure 1E,F and Figure 3C as well as in Table 1, Si–OH–Al groups in zeolite HMFI are heterogeneous. ^29^Si MAS NMR of this zeolite (Figure 4C) shows intensive signal of Si(0Al), less intensive signal of Si(1Al), and a weak signal of Si(2Al). Therefore, besides strongly acidic (SiO)_3_Si–OH–Al(OSi)_3_, small amount of less acidic (AlO)(SiO)_2_Si–OH–Al(OSi)_3_ is present. Another reason for the heterogeneity of OH groups in HMFI may be the presence of Si–OH–Al of various bridge geometries. There are 12 crystallographically nonequivalent positions of T atoms in MFI lattice; moreover, both monoclinic and orthorhombic forms coexist in this zeolite. The consequence of this is the presence of 21 very narrow Si(0Al) lines in ultra-high resolution ^29^Si MAS NMR spectrum of silicalite reported by Fyfe et al. [71]. Our NMR spectrum of zeolite MFI (Figure 4C) also shows the splitting of Si(0Al) signal into two lines. The T-O-T bridge angle is in the limits between 143 and 175° [8]. The problem is that the location of Al atoms in MFI framework is not known; however, it is not excluded that the Si–OH–Al bridges of various bridge geometries may exist, and it may be one of reasons for the presence of hydroxyls of various acid strengths in zeolite HMFI observed in our study. As mentioned above, another reason for the heterogeneity may be the presence of hydroxyls of various numbers of Al atoms in the vicinity.

The acid properties of zeolites HMFI were subject of very intensive studies. The problem of homogeneity and heterogeneity of Si–OH–Al was discussed in the literature, and some arguments supporting the thesis of heterogeneity and some others against this thesis were discussed. One argument supporting this thesis was the splitting of Si–OH–Al band in HMFI of Si/Al of Si/Al above 50 in the spectra recorded at low temperatures (ca. 170 K) [72]. Another argument was the shift of IR band of free hydroxyls in the spectra recorded upon desorption of ammonia at various temperatures [73]. On the other hand, catalytic activity of HMFI increased linearly with the amount of framework Al [74], which suggested the uniformity of Si–OH–Al. Very interesting results and valuable discussion concerning interaction of OH groups in HMFI with CO and N_2_ were presented by Chakarova at al. [58] Their results supported the thesis of homogeneity of Si–OH–Al in HMFI.

The results obtained in the present study suggest the heterogeneity of Si–OH–Al in zeolite HMFI. It seems that the reason for the disagreement in the interpretations concerning the heterogeneity of Si–OH–Al in MFI may be the differences in the amount and location of Al in the framework. In zeolites of low Al content, only one kind of strongly acidic (SiO)_3_Si–OH–Al(OSi)_3_ may exist, whereas in zeolites containing more Al (lower Si/Al), not only the above-mentioned Si–OH–Al but also less acidic (AlO)(SiO)_2_Si–OH–Al(OSi)_3_ may be present. Therefore, in zeolites of high Si/Al, Si–OH–Al may be homogeneous, but in zeolites of lower Si/Al, they may be heterogeneous. Our zeolite MFI has rather low Si/Al (Si/Al = 20), so it may be one of the reasons for the heterogeneity of Si–OH–Al in our zeolite. As mentioned above, another reason for heterogeneity of Si–OH–Al in zeolites MFI may be also the presence of Si–OH–Al of various bridging bond angles. The location of Al atoms in MFI framework is not known, but it may be supposed that it depends on the composition and synthesis conditions. It may happen that in some zeolites all Al atoms may take the same T positions, generating Si–OH–Al of the same bridge angle and therefore of the same acid strength. On the other hand, in some other zeolites Al atoms may occupy different T positions generating Si–OH–Al of different Si–OH–Al bridge angles and therefore various acid strengths. This may be the reason why some authors observed homogeneity and some others observed heterogeneity of Si–OH–Al in MFI.

Even though zeolite BEA is very interesting as catalyst for bulky molecules and its acid properties were studied by several authors [75,76,77,78], there are no results concerning the problem of heterogeneity of Si–OH–Al. The results obtained in our study evidence the heterogeneity of these hydroxyls (Figure 1G,H and Figure 3D), and in Table 1 we consider that the reasons why Si–OH–Al in HBEA are heterogeneous are similar to those in the case of HMFI. The ^29^Si MAS NMR spectrum of zeolite BEA shows signals of Si(0Al), Si(1Al), and a weak one of Si(2Al). Therefore, both strongly acidic (SiO)_3_Si–OH–Al(OSi)_3_ and small amount of less acidic (AlO)(SiO)_2_Si–OH–Al(OSi)_3_ are present. Moreover, nine non-equivalent T atoms positions are present and T-O-T bridged angles are in the range 148–155.9° [79]. Like for zeolite MFI in our NMR spectrum, the signal of Si(0Al) shows two components indicating the presence of non-equivalent Si positions. Therefore, both reasons—the presence of Si(1Al) and Si(2Al) and presence of T-O-T bridges of various geometries—may be responsible for heterogeneity of Si–OH–Al in zeolite HBEA.

Generally, the fact that the heterogeneity of Si–OH–Al in some zeolites and homogeneity in other ones constitutes evidence that the properties of zeolitic hydroxyls are strictly related to the properties of framework Si and Al. It concerns not only the number of Al near the bridge but also the bridge geometry.

## 4. Materials and Methods

### 4.1. Materials

The parent Na-Y zeolite was synthesized according to the instructions in [80]. Framework Si/Al calculated from ^29^Si MAS NMR results was 2.5. Ion exchange was performed by mixing NaY zeolite powder with water solution of NH_4_NO_3_. Two grams of zeolite NaY were mixed with 0.2 dm^3^ of 4 × 10^−3^ mol/dm^3^ solution at room temperature under stirring conditions. Stirring time was 15 min. Afterwards, the sample was centrifuged, washed with distilled water four times, and dried at 70 °C in an oven for 18 h. The sample of exchange degree 11% was obtained.

Zeolites HFAU of Si/Al = 31 were produced by Zeolyst (CBV 760). Zeolites HMFI and HBEA were purchased from Evonic (Essen, Germany) and from RIPP (Beijing, China), respectively. The values of Si/Al of these zeolites provided by the producers were 20 (HMFI) and 25 (HBEA), respectively.

Ammonia was purchased from Air Products, Allentown, PA, USA. Gaseous nitrogen was gained by the evaporation of liquid nitrogen (Air Products, Allentown, PA, USA).

### 4.2. IR Studies

Prior to IR experiments, zeolites wafers (20–30 mg/cm^2^) were evacuated in situ in an IR cell at 720 K for 1 h. The spectra were recorded with a NICOLET 6700 spectrometer (Thermo Scientific, Cambridge, MA, USA) with the spectral resolution of 1 cm^−1^. NH_3_ was adsorbed at 400 K and subsequently desorbed at various temperatures (415–505 K). The cell for IR studies (with CaF_2_ windows) was constructed in such a manner that the sample could be heated until 800 °C (in needed) or cooled to ca. −140 °C without moving the wafer. In order to adsorb nitrogen, the cell was cooled by immersing with nitrogen the kaolin wool wrapping the cell. This very simple method presented the possibility of cooling the sample till −140 °C. The doses of nitrogen were adsorbed at ca. 170 K until the band of free OH lost ca. 80–90% of its original intensity. The nitrogen pressure inside the cell was not controlled. The cell was cooled to ca. 170 K with liquid nitrogen.

### 4.3. NMR Studies

^29^Si MAS NMR spectra were recorded using Bruker Avance III 500 MHz spectrometer (Billerica, MA, USA) using a resonance frequency of 99.4 MHz. Samples were packed to 4 mm rotor and spun at a speed of 8 kHz. The spectrum was recorded using pi/3 pulse (5.8 µs), recycle delay 24 s, and 9216 scans.

## 5. Conclusions

Nitrogen was proposed to be a probe molecule for the IR studies of the heterogeneity of acidic Si–OH–Al groups in zeolites. The advantages of nitrogen, if comparing it with commonly used CO, are that the frequency shift of the band of Si–OH–Al interacting with nitrogen by hydrogen bonging is relatively low (70–130 cm^−1^) and the shifted OH…N_2_ band does not overlap the bands of ammonium ions. Therefore, it is possible to investigate the spectra of hydroxyls interacting with N_2_ in the zeolites, in which ammonia was adsorbed and subsequently desorbed at increasing temperatures. The red frequency shift of the band of OH…N_2_ with ammonia desorption temperature constitutes evidence of the heterogeneity of Si–OH–Al. This is because the less acidic hydroxyls of lower Δν_OH…N2_ release ammonia at lower temperatures.

Si–OH–Al groups in zeolites NaHY, HMFI, and HBEA were found to be heterogeneous, and Si–OH–Al in zeolite HFAU (of Si/Al = 31) were found to be homogeneous. The heterogeneity of Si–OH–Al in NaHY was explained by the presence of hydroxyls of various numbers of Al atoms in close vicinity. According to ^29^Si MAS NMR studies, Si(1Al), Si(2Al), and Si(3Al) are present and correspondingly hydroxyls of various acidities may be represented by the following formula: (SiO)_3_Si–OH–Al(OSi)_3_, (AlO)(SiO)_2_Si–OH–Al(OSi)_3,_ and (AlO)_2_(SiO)Si–OH–Al(OSi)_3_. On the other hand, in highly siliceous HFAU only (SiO)_3_Si–OH–Al(OSi)_3_ are present and homogeneity of OH groups was observed.

Heterogeneity of Si–OH–Al in zeolites HMFI and HBEA can be explained by the presence of (SiO)_3_Si–OH–Al(OSi)_3_, and small amounts of (AlO)(SiO)_2_Si–OH–Al(OSi)_3_ of various acidities and also by the presence of Si–OH–Al of various bridge geometries.

## Figures and Tables

**Table 1:** The frequencies of IR bands of Si–OH–Al free and interacting with nitrogen in zeolites in which ammonia was sorbed and subsequently desorbed at various temperatures.

	NH_3_ Desorption Temperature	ν_OH_		ν_OH,,,N2_	Δν_OH,,,N2_
		400 K	170 K		
NaHY	418 K	3646	3656	3580	76
	433 K	3645	3654	3572	82
	443 K	3644	3653	3568	85
	458 K	3644	3653	3564	91
	without NH_3_	3641	3650	3558	92
HFAU	473 K	3626	3634	3496	138
	483 K	3626	3634	3495	139
	505 K	3627	3634	3496	138
	without NH_3_	3627	3633	3495	138
HMFI	473 K	3611	3619	3507	112
	483 K	3610	3620	3500	120
	503 K	3608	3619	3496	125
	518 K	3607	3619	3495	124
	without NH_3_	3607	3619	3495	124
HBEA	443 K	3617	3627	3515	112
	463 K	3615	3625	3511	114
	483 K	3615	3619	3500	119
	503 K	3610	3618	3491	127
	without NH_3_	3607	3613	3486	127

## Data Availability

Not applicable.

## Sample Availability

Samples that are zeolites of NaHY-, HFAU-, HMFI-, and HBEA-type structures are available from the authors.
